# Feldspar-Modified Methacrylic Composite for Fabrication of Prosthetic Teeth

**DOI:** 10.3390/ma16103674

**Published:** 2023-05-11

**Authors:** Zbigniew Raszewski, Julita Kulbacka, Daria Pakuła, Dariusz Brząkalski, Robert E. Przekop

**Affiliations:** 1 R&D, SpofaDental, Markova 238, 506-01 Jicin, Czech Republic; 2 Department of Molecular and Cellular Biology, Faculty of Pharmacy, Wroclaw Medical University, Borowska 211A, 50-556 Wroclaw, Poland; julita.kulbacka@umed.wroc.pl; 3 Department of Immunology, State Research Institute Centre for Innovative Medicine, 08410 Vilnius, Lithuania; 4 Faculty of Chemistry, Adam Mickiewicz University in Poznan, 61-614 Poznan, Poland; darpak@amu.edu.pl; 5 Centre for Advanced Technologies, Adam Mickiewicz University in Poznan, 61-614 Poznan, Poland; dariusz.brzakalski@amu.edu.pl

**Keywords:** acrylic teeth, methacrylic polymer, feldspar silane, surface modification, connection between teeth and denture base, compressive strength, cytotoxicity, biomaterials

## Abstract

This study was aimed at investigating poly(methyl methacrylate) (PMMA), modified with a silanized feldspar filler at 10 wt.% and 30 wt.%, as a dental material system for the production of prosthetic teeth. Samples of this composite were subjected to a compressive strength test, three-layer methacrylic teeth were fabricated with the said materials, and their connection to a denture plate was examined. The biocompatibility of the materials was assessed via cytotoxicity tests on human gingival fibroblasts (HGFs) and Chinese hamster ovarian cells (CHO-K1). The addition of feldspar significantly improved the material’s compressive strength, with neat PMMA reaching 107 MPa, and the addition of 30% feldspar raising it up to 159 MPa. As observed, composite teeth (cervical part made of neat PMMA, dentin with 10 wt.%, and enamel with 30 wt.% of feldspar) had good adhesion to the denture plate. Neither of the tested materials revealed any cytotoxic effects. In the case of hamster fibroblasts, increased cell viability was observed, with only morphological changes being noticed. Samples containing 10% or 30% of inorganic filler were determined to be safe for treated cells. The use of silanized feldspar to fabricate composite teeth increased their hardness, which is of significant clinical importance for the duration of use of non-retained dentures.

## 1. Introduction

Prosthetic teeth have been in use for millennia now, the oldest examples dating back to ancient times, where among different cultures, bones, animal teeth, and various metals were utilized for the fabrication of prosthetics [1]. In modern dentistry, naturally, due to the requirements for ensuring biocompatibility with no danger of acute toxicity or infection being induced upon the introduction into the patient’s tissue, on top of easy shaping and tailoring up to the required application and consistency of properties (mechanical, tribological, optical), synthetic materials and composites thereof have prevailed. Properties such as biological inertness, fracture resistance, and adhesion to either the patient’s tissue or other prosthetic components (e.g., a denture plate) are essential. One of the most popular materials for non-denture dentures is PMMA, which has very good optical properties, and it is easy to pigment in a colour corresponding to the natural colour of the tooth [2,3].

However, it is a soft material that wears down very quickly, both under the influence of chewing forces or in contact with natural antagonistic teeth. After about two years, the resin dentures are subject to about a 2–3 mm loss. Changes in the height of the teeth resulting from the abrasion of their surface change the vertical dimension of the occlusion, which can lead to craniofacial disorders, reduce chewing efficiency, cause masticatory muscle fatigue, increase patient discomfort, and worsen the aesthetics. Although there are different materials on the market used to make teeth for removable dentures (porcelain, composite), PMMA is the most frequently used material. Compared to porcelain teeth, teeth made of poly(methyl methacrylate) are less brittle, have better connection to the denture base material, are easier for occlusal adjustments and repolishing, have a more natural appearance, and create less chewing noise [4,5,6,7]. Various types of fillers can be added to the material to improve its mechanical properties, which can increase its hardness by causing abrasion [8,9,10]. Some authors believe that modified teeth have less adhesion to the denture plate [11]. Therefore, having the possibility to fabricate layered composite teeth, our team decided to make such tests. The cervical layer was made of neat poly(methyl methacrylate) to provide better connection of the tooth to the denture plate. From our previous work, it appears that fillers must be silanized on the surface so that it is possible to create a chemical bond between the inorganic part and the methacrylic resin [12]. In order to check the correctness of the operation of such teeth, several tests have to be performed. One of the tests determining the hardness of a material is its resistance to compressive strength [13,14,15]. An additional ISO 22112:2017 [16] standard stipulates that each material used to make a denture embedded in the plate must create an appropriate connection between the prosthesis and the tooth. The first test to be performed with a new medical device is the cytotoxicity test, which indicates the safety of the material in contact with body tissues [17,18,19,20,21].

This work is a development of a previous one, in which our team worked on a new acrylic material for teeth with increased resistance. The first article published in 2020 examined the basic mechanical parameters of an acrylic material modified with salinized feldspar [12]. This research aimed to capture the influence of different silanized feldspar loading on a composite material’s compressive strength resistance and the connection between the denture plate and the teeth. For verification of the biocompatibility, cytotoxicity tests were also performed on the cortices of human fibroblasts and hamster ovarian cells.

The thesis put forward at the beginning of this study is that the obtained material will not be cytotoxic, it will have adequate resistance to crushing, so as to withstand the pressure generated during the food mastication, and proper adhesion do the denture base. This study is a continuation of our previous work, where the maximum displacement, elastic modulus, Isolde impact resistance, and Brinell hardness were tested for the same feldspar filling system.

## 2. Materials and Methods

### 2.1. Materials and Their Preparation

Silanized feldspar, Microspar 1351-600, was obtained from Mineral Engineers (Magdeburg, Germany). Colacryl D80 FC, poly(methyl methacrylate) (PMMA) resin powder was obtained from Lucite international, England (Billingham). Human gingival fibroblasts (HGFs) were isolated from healthy gingival tissues according to a procedure described elsewhere [22]. Chinese hamster ovarian cells (CHO-K1) were obtained from our own culture in the molecular and cellular biology laboratory (Wroclaw Medical University, Wroclaw, Poland). For cell cultivation, Dulbecco Modified Medium (DMEM, Sartorius, Kostrzyn Wlkp, Poland) and HAM’s F10 Medium (Merck Life Science, Poznan, Poland), 10% FBS (fetal bovine serum, HyClone, Logan, UT 84321, USA), and 24-well plates (Sarstedt, Nümbrecht, Germany) were used. PrestoBlue^®^ assay (Thermo Scientific, Warsaw, Poland) was used for a cell viability study.

The thorough preparation of the cell cultures and the tests performed were described in detail in the previous work of our team [22,23]. The experiments were performed as follows: cells were seeded on 24-well plates (Sarstedt, Germany) with sterile utensils. PrestoBlue assay (Thermo Scientific, Warsaw, Poland) was performed after 24 h of exposition. Microscopic observations were also performed after 24 h using a Leica inverted microscope (DMi1, Watzlar, Germany).

**Figure 1:**
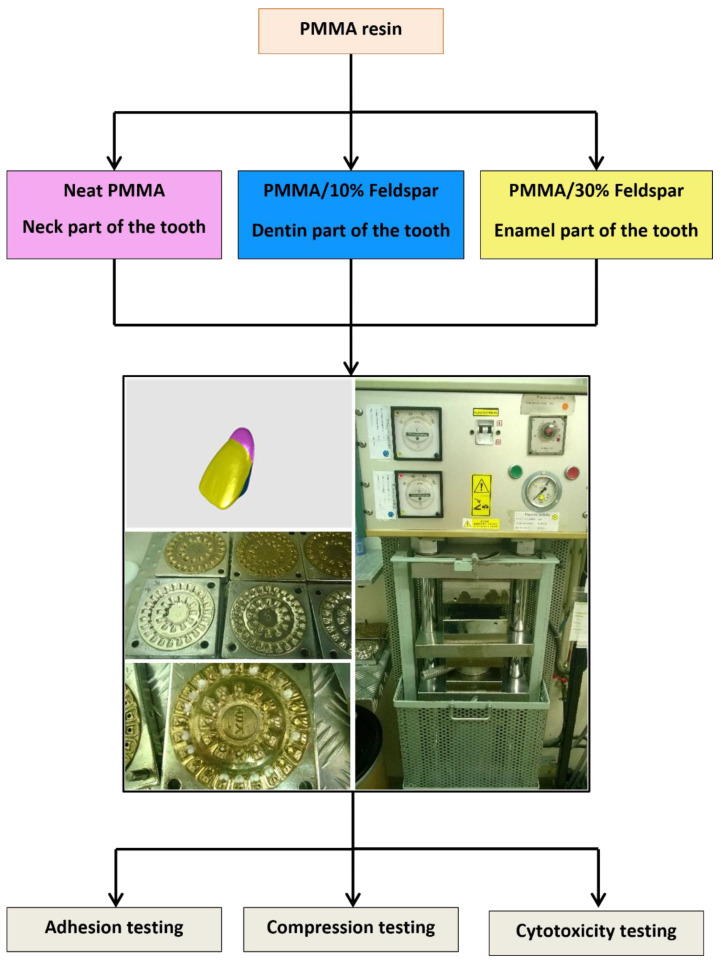
Graphical abstract for the tests performed in this study.

Silanized feldspar was added to PMMA resin powder to prepare 0%, 10%, and 30% by weight samples by mixing the powders in a ball mill (Jezirska Porcelanaka, Czech Republic) for 1 h. The PMMA powder together with the appropriate amount of feldspar was weighed on a scale to obtain 1 kg of the final powder. What was also added to the material was 1% titanium oxide (KRONOS 9900 white pigment, Middlesex County, MA, USA) and 0.5–0.12% yellow (V Ferrox Yellow 02), brown, and red (V Ferrox Reds 05) colours (all iron oxide dyes, Ferrox Tamilnadu, St.Thomas Mount, India). Two kg of ceramic balls with a diameter of 20–40 mm were added to the ball mill. After the mixing process, the balls were separated from the powder using a 100-micron sieve. The coloured powder was poured into polyethylene containers and used for further research. The samples prepared in this way were mixed with the methacrylic monomer Superacryl Plus (SpofaDental, Jicin, Czech Republic) in a weight proportion of 2:1 powder/liquid monomer. After mixing the powder with the liquid, the whole mixture was stored in a glass vessel under a cover to prevent the methyl methacrylate from evaporating. When the acrylic resin did not stick to the metal spatula, after approx. 15 min the material in the form of a soft dough was placed in unfolded steel moulds of the appropriate shape, depending on the type of testing method to be performed. The design of the experiment and the test pieces are summarized in Figure 1.

For compressive strength tests, the material was placed in a cylindrical mould with a 4 mm diameter and a height of 10 mm. The top and bottom of the mould were covered with polyester foil and a 10-mm-thick metal plate. During the next step, the moulds with methacrylic resin inside were placed into the hand press Mestra (Taleres, Spain) under 2000 kg pressure for 10 min to obtain the final shape and remove the excess material. The material was polymerized in press LHP (DMI Instruments, Sydney, Australia) at 130 °C, for 6 min under 12 bar pressure, in accordance with the manufacturer’s instructions. After the curing process was completed, the samples were allowed to cool, the moulds were opened, and the composite resin specimens were removed. A total of 30 samples were made, 10 for each feldspar concentration.

**Figure 2:**
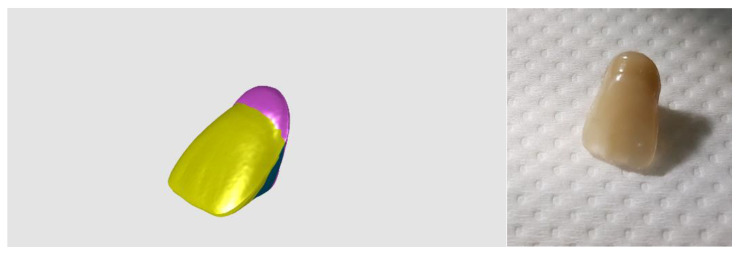
A 3D model of a 3-layer composite tooth (**left**) and the final composite tooth (**right**); colours were used at the modelling stage to represent the model parts made of different material compositions. Red colour for the neck part made from neat PMMA, blue part for dentin part (PMMA/10% feldspar), and yellow colour for the enamel layer (PMMA/30% feldspar).

**Figure 3:**
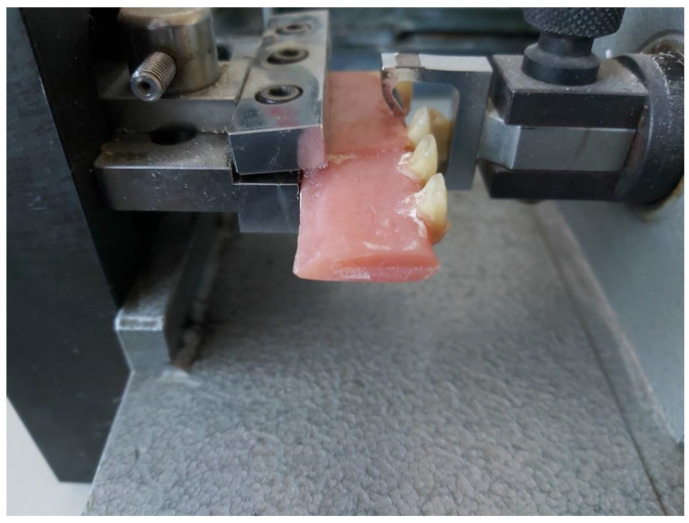
The examination of the connection of composite teeth to the denture plate.

To examine the connection between the teeth and the denture plate, the first step was to prepare samples of three-layered composite teeth. The tooth model is presented in Figure 2, the red–blue–yellow colour set being applied only for the 3D model during the modelling stage, while at the specimen preparation stage, the standard enamel colour set was used. For the purpose of sample preparation, three-part tooth forms of the upper incisor were used (Fonak, Pardubice, Czech Republic). At the beginning, a methacrylic base containing Colacryl D 80FC (with proper colours) was mixed with a Superacryl Plus (SpofaDental, Jicin, Czech Republic) monomer (mixing ratio 2:1 by weight) and, after 15 min, at the dough stage it was placed in the mould. This material was used to make the cervical part of the tooth, and the piece was dark-yellow coloured. The moulded material was polymerized in a heating press LHP (DMI Instruments, Sydney, Australia) at 130 °C for 3 min under 12 bar pressure, the same polymerization conditions being used for each of the three polymerization stages. After opening the moulds, a yellow dentin part was applied to obtain 10% silanized feldspar (made by mixing the powder and liquid in a 2:1 weight ratio), and the mould was closed. The whole piece was placed back in the press for a period of 3 min. After the neck and dentin parts had been cured, the mould was re-opened, and the material containing 30% silanized feldspar was placed in it as the enamel part (white/transparent colour). Three colours were chosen to be able to trace the connection between the different layers in the teeth (enamel, dentin, and cervical sections) when examining the connection to the denture plate (Figure 2 and Figure 3).

The whole model was placed in the press for the third time for 6 min. The mould with the tooth model was cooled down for 10 min on a metal plate and opened. The removed composite prosthetic teeth were used to examine the connection between the denture plate and the PMMA-based composite.

### 2.2. Materials Characterization

Fourier transform infrared (FT-IR) spectra were recorded on a Nicolet iS 50 Fourier transform spectrophotometer (Thermo Fisher Scientific, Warsaw, Poland) equipped with a diamond ATR unit with a resolution of 0.09 cm^−1^.

Differential scanning calorimetry (DSC) was performed using a NETZSCH 204 F1 Phoenix (Netsch Selb, Bayern, Germany) calorimeter. Samples of 6 ± 0.2 mg were placed in an aluminium crucible with a punctured lid. The measurements were performed under a nitrogen atmosphere in the temperature range of 20–200 °C and at a 10 °C/min heating rate.

### 2.3. Mechanical Analysis

For the compressive strength tests, the obtained cylinder-shaped specimens were used, five pieces for each measurement, and subjected to a compression test after 24 h of storage in distilled water in a laboratory drier at 37 °C. The next 5 samples at each feldspar concentration were stored for 7 days under the conditions described above. The compressive strength was tested on a Shimadzu machine Autograph Universal Testing Machine Series AG-5 kN (Shimadzu, Kyoto, Japan) at the testing speed of 0.5 mm/min, in accordance with the literature reports on such analysis [2].

For testing the shared bond strength between the denture base and the composite tooth, the research was performed in accordance with the ISO 22112:2017. A strip of modelling wax (Ceradent, SpofaDental, Jicin, Czech Republic) was fixed to the front part of the metal plate described in the standard. Tested incisors with feldspar (5 pieces) polymerized as before, were attached to this wax. The whole was placed in a polymerization flask and covered with Snow White Plaster 2 (Kerr, Scafati, Italy), so that the tooth surfaces were covered with plaster. After the gypsum had set, the wax was boiled out with hot water. In the last place, acrylic resin was applied to the denture base material Superacryl Plus (SpofaDental) and polymerized according to the manufacturer’s instructions. The device Famed 3 (Lapka, Prague, Czech Republic) was used to test the connection of the composite teeth with the denture plate (Figure 3).

### 2.4. Cytotoxicity Tests

Human gingival fibroblasts (HGFs) and Chinese hamster ovarian cells (CHO-K1) were used to conduct the experiments in this study. The HGFs were cultivated in Dulbecco Modified Medium (DMEM, Sartorius, Kostrzyn Wlkp, Poland), and the CHO-K1 cells were cultivated in HAM’s F10 medium with the addition of 1% antibiotic Penicillin (Merck Life Science, Poznan, Poland) and 10% FBS. The thorough preparation of cell cultures and the tests performed were described in detail in the previous work of our team [21,22]. The experiments were performed as follows: cells were seeded on 24-well plates, with all operations performed with sterile tools. A cell viability assay was performed after 24 h of exposition. Microscopic observations were also performed after 24 h using a Leica inverted microscope (DMi1, Watzlar, Germany).

### 2.5. Statistical Analysis

The results were statistically analysed using a one-way analysis of variance at a significance level of 0.05, as a reference using a sample of PMMA resin not modified with fillers. In addition, a post hoc analysis was performed by using Tukey’s HSD test (using a free test calculator provided by Astatsa, San Jose, CA, USA).

## 3. Results and Discussion

### 3.1. FT-IR Spectroscopy

**Figure 4:**
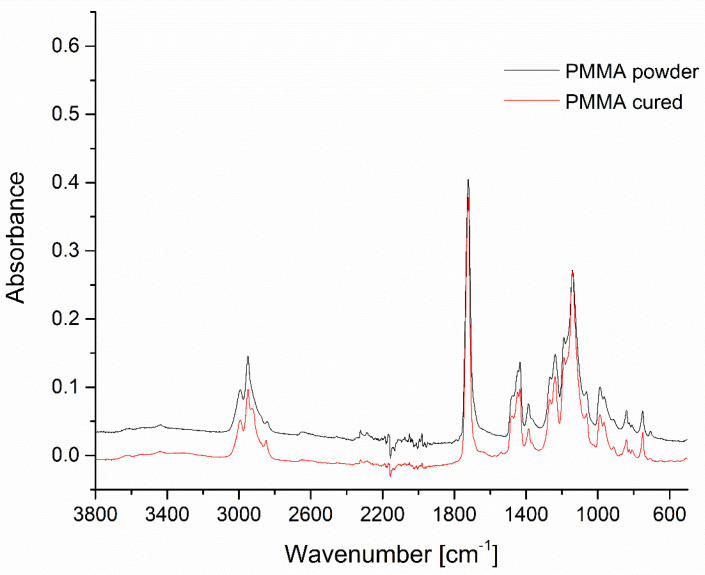
FT-IR spectra of PMMA powder and cured PMMA compared. The spectra are offset for clarity.

The filler materials, i.e., Microspar 1351-600 silanized feldspar and poly(methyl methacrylate) resin powder, as well as the prepared composites were studied by means of FT-IR spectroscopy. The spectrum of the Colacryl D80 FC powder matched the typical characteristics of PMMA, with a strong carbonyl stretching vibration maximum at 1722 cm^−1^, C-H deformations in the 1470–1380 cm^−1^ range, and C-O stretching vibrations in 1266–1140 cm^−1^ (Figure 4) [24,25].

No bands in the 1500–1600 cm^−1^ range were visible, proving the absence of methacrylate monomers. The spectrum of the cured two-component system (Colacryl D80 FC with the liquid monomer in a 2:1 weight ratio) was virtually identical to the neat Colacryl D80 FC, as after curing, the system became a uniform PMMA material with no spectral differences visible.

**Figure 5:**
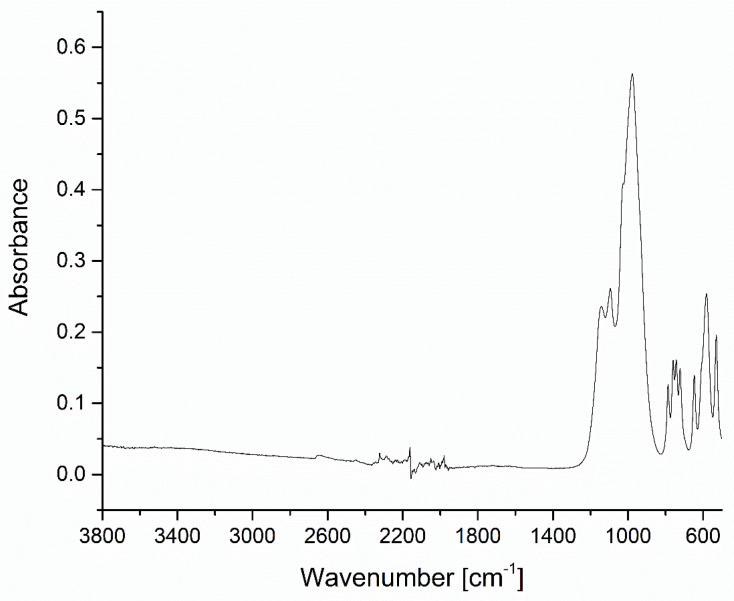
FT-IR spectra of silanized feldspar.

Silanized feldspar was characterized by a typical spectrum of neat feldspar, with peak maximums of 977, 1027, 1094, and 1142 cm^−1^, assigned to different types of Si-O asymmetric stretching vibration modes, which was in a good agreement with the findings of Bosch-Reig et al. (Figure 5) [26]. No bands typical for organosilanes were visible, therefore silanization was likely performed by coating the material with a SiO_2_ nanolayer.

**Figure 6:**
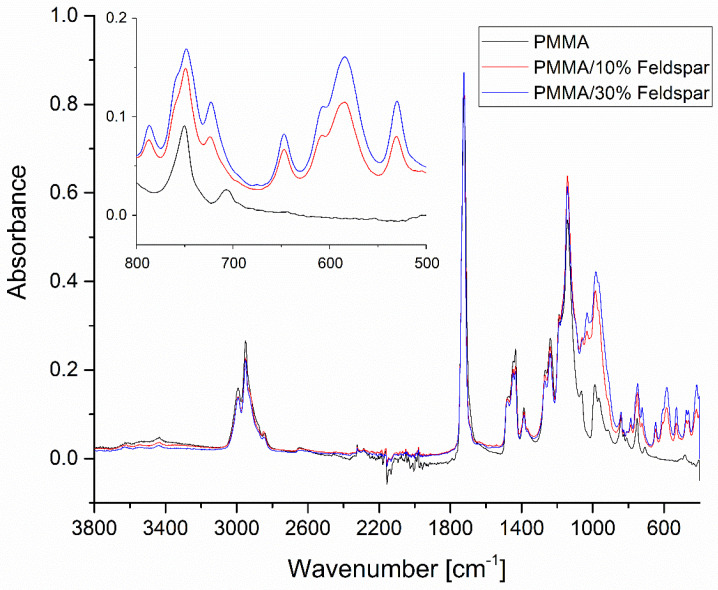
FT-IR spectra of PMMA composites filled with 0, 10, and 30% of silanized feldspar.

The FT-IR spectra of the composite systems of 10% and 30 wt.% feldspar-filled PMMA were convolutions of the abovementioned spectra, with the appropriate bands assigned to feldspar increasing in intensity together with the filler loading (Figure 6). The good agreement between the filler loading and the intensity of the abovementioned band may be a basis for a semi-quantitative composition assessment of feldspar–PMMA composites of unknown loading or as a tool for studying filler distribution homogeneity in the matrix. A band especially useful for such an operation was visible at ~580 cm^−1^, where no absorption was present for neat PMMA.

### 3.2. Differential Scanning Calorimetry

**Figure 7:**
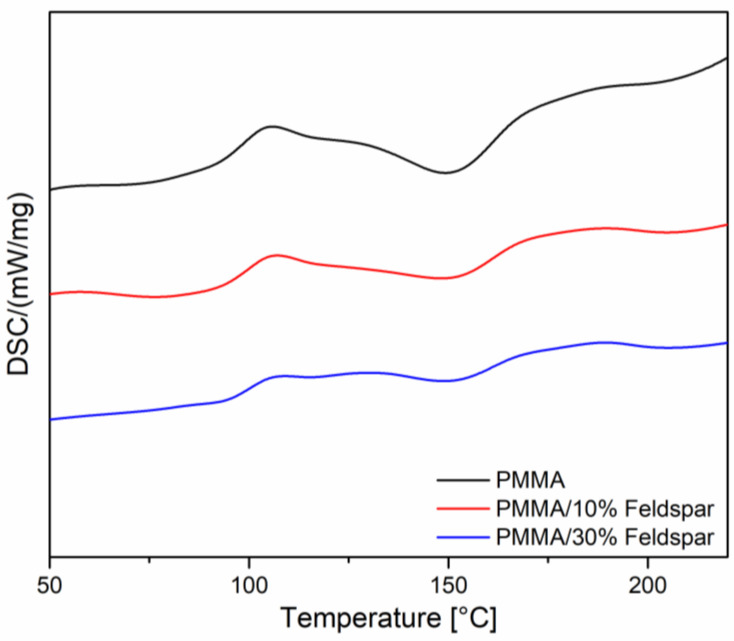
FT-IR spectra of PMMA and PMMA/feldspar composites.

DSC analysis allowed for the determination of the characteristic phase transitions and thermal events within the PMMA. On the basis of the obtained heating curves, the temperature of the glass transition (T_g_) was determined and observed to be within 90–110 °C for all the samples (Figure 7). For the reference sample, the T_g_ peak was 105.8 °C. The addition of the filler caused minute changes in this feature, resulting in the rise of T_g_ by 1.2 °C and 2.6 °C for 10% and 30% loading, respectively. This effect can be explained by a weak interaction between the filler and polymer, causing a very slight reduction in the polymer network freedom. Bosch-Reig et al. observed a similar behaviour for PMMA [26]. The decreasing peak area for the composites was to be expected, as the filler in question is inert upon heating in the studied temperature range. In the 130–170 °C range, an exothermic event was observed, being the most pronounced for the neat PMMA. It may be assigned to deeper crosslinking of the remaining methacrylate oligomers, as the material was cured under the temperature of 130 °C, according to the manufacturer’s instructions.

### 3.3. Mechanical Analysis

**Figure 8:**
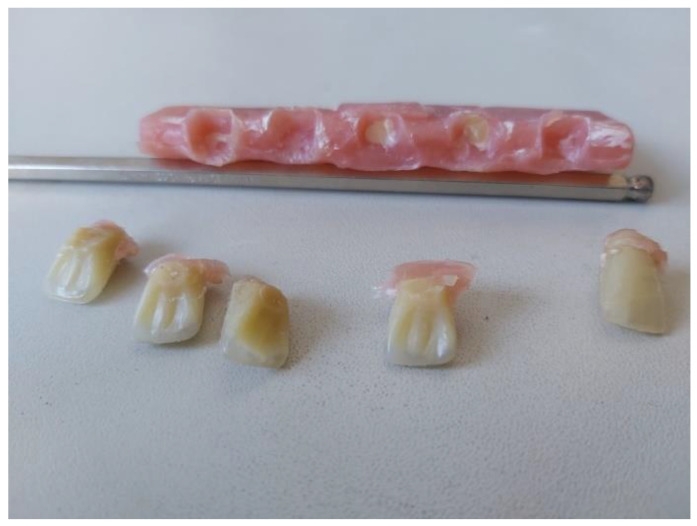
The representation of the composite teeth separation from the prosthetic plate, fractured parts of the teeth visible in the methacrylic mass, as well as methacrylic residues on the surface of the teeth.

The results obtained in this study are presented in the following Table 1, Table 2 and Table 3. A graphical representation shows the connection of the teeth with the denture plate (Figure 8).

The compressive strength of neat PMMA is dependent on the curing conditions thereof but is often below 100 MPa [5]. In this work, a higher value was obtained because the material was polymerized at a higher temperature of 130 °C, which is a value higher than the PMMA glass transition temperature (100–110 °C), which ensures better cross-linking resistance and lower residual monomer content.

The addition of feldspar to methacrylate resin increases its compression resistance. This was most evident for a sample containing 30% of this filler (159.7 ± 4.1 MPa). After seven days of conditioning, a slight decrease in the compressive strength by about 10% was observed for all the materials, which was due to water absorption into the materials, causing hydrolysis and plasticization of the polymer. It is a property commonly found in measuring the fracture toughness of all (meth)acrylics [2,5,12].

The addition of feldspar to the PMMA material increased its resistance to compressive forces. The compressive strength values of 100–120 MPa obtained in these tests are consistent with the work of Szabelski et al. [13] who tested a system with barium sulphate as an inert filler. It is known that this material does not chemically combine with PMMA (it cannot be successfully subjected to the silanization process). Similar conclusions regarding the increased resistance to abrasion and the hardness of methacrylic teeth with inorganic fillers were also drawn by Mello (2009) [4].

The results of examining the connection of the teeth with the prosthetic plate after 24 h and 7 days of conditioning in water at 37 °C are presented in Table 3.

According to the requirements of ISO 22112:2017, the results indicate that the developed methacrylic teeth had good adhesion to the denture plate. In all cases, a cohesive type of connection was observed (Figure 8). Part of the tooth remained on the surface of the denture plate, and part of the methacrylic base material was attached to the tooth. There was no delamination at the interphase of these two. There was no statistical difference between the combination of teeth after 24 h and 7 days. The standard does not require specifying the strength, only examining the type of the tear itself, whether it is adhesive or cohesive. A sample of 5 teeth all met the above-mentioned requirements and were, according to the recommendations of the standard, representative. The values of the strength of the connection between individual teeth were compared after both 24 h and 7 days, which indicates the durability of this connection over a certain period of time. However, it requires checking over a longer period, because the prosthesis as such is used even for 2–3 years. A complex three-layer structure, which in the cervical part is made of PMMA and the enamel part is a mixture of 30% silicate-based filler, has proper adhesion to the denture plate made of methacrylic resin. Similar bond values of 20–30 N between acrylic teeth available on the market and the denture plate were also obtained by other authors, for the same number of tested samples [27,28,29,30]. However, special attention should be paid to the preparation of the tooth surface by extending its surface with burs and activating it with coupling agents, which has already been widely discussed in the literature on the subject [6,28,29,30].

During this work, the authors used a thermal method for removing wax and washing the plaster surface with an active agent and hot water to obtain a proper connection between the teeth and the denture plate. Additionally, the tooth surface was rinsed 3 times for 30 s with monomer [31,32]. The connection of the teeth with the denture plate was the best in the case of the thermally polymerized material [33]. Therefore, in this study, the teeth were made late with the heat curing Superacryl Plus methacrylic resins for removable dentures. These results are also consistent with the research of Prpić V at al [32], where the lowest adhesion values were recorded for self-cure materials, namely 3.37 ± 2.14 MPa to 18.10 ± 2.68 MPa for thermally polymerized materials. In this study, a strong connection of the new teeth with the denture plate was obtained; the adhesive force needed to detach the tooth from the denture plate was 26.57 ± 1.28 [N]. However, long-term examinations are necessary to fully establish the connection of the denture plate with the teeth. As was shown in the literature, the connection may weaken over time by about 10–15% [34,35].

### 3.4. Biological Compatibility Study

**Figure 9:**
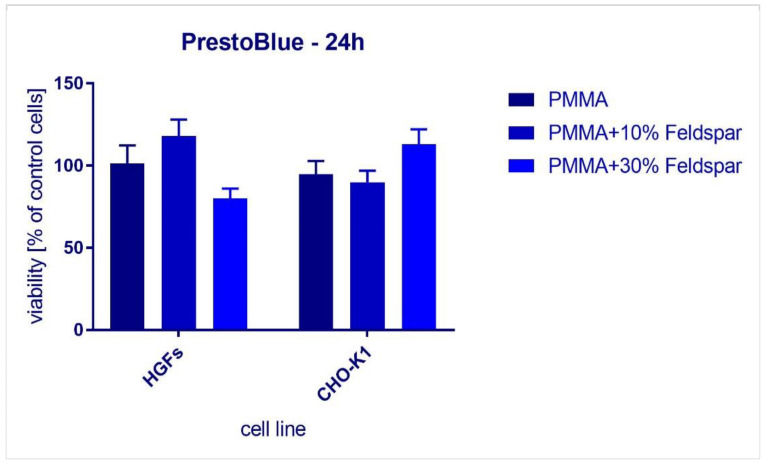
The effects of 24 h exposure to resins on human (HGFs) and hamster ovarian cells (CHO-K1).

The success of prosthetic rehabilitation in the case of a non-permanent denture depends on proper conduct to obtain the comfort, functionality, and aesthetics of the established restoration. Choosing materials to replace the missing structures, especially the teeth, is a critical clinical problem for both the dentist and the patient [5]. However, despite the abovementioned characteristics, the selected components must be non-cytotoxic towards the tissues they are about to be in contact with. The compatibility of the materials with biological tissues was assessed with the use of a standard cytotoxicity assay kit utilizing human gingival fibroblasts (HGFs) and Chinese hamster ovarian cells (CHO-K1) (Figure 9). The samples did not reveal cytotoxic effects. In the case of CHO-K1, we observed increased cell viability. Samples containing 10% or 30% of inorganic filler were determined to be safe for the treated cells, in particular for gingival fibroblasts. Saavedra et al. [35]. indicated that feldspar powder with very good granulation has cytotoxic properties against human bronchial epithelial cells (HBEC3-KT), THP-1 macrophages, and a HBEC3-KT/THP-1 co-culture. Other authors reported similar results. Feldspar was slightly cytotoxic to NR8383 cells, as stated by Attik et al. (2008) [36]. In our case, however, these particles were well bound within the PMMA matrix, therefore they were not in the form of a powder. In addition, PMMA, cured at a high temperature, is also a biologically compatible material. However, dental technicians who are fabricating a new denture may be exposed to adverse effects from feldspar powder. Therefore, it seems necessary to comply with health and safety rules considering working with protective masks and laboratory hoods connected to the working table [37].

However, the conducted research has several limitations. Firstly, before a composite or acrylic material is approved for sale according to the normative requirements, it must undergo many tests. These tests are only in their early stages. In subsequent stages, it will be necessary try to use other cell cultures for cytotoxicity studies. In the following stage these materials in terms of irritation (skin tests) and within the mucosa of the oral cavity and implantation will be examined. It will also be necessary to carry out tests on colour stability, its change under the influence of use in colouring solutions such as coffee, tea, or red wine, and long-time adhesion between the denture base and new material.

To investigate the link between silanized feldspar and PMMA at the molecular level, further studies using electron microscope techniques will be necessary. In our previous work, it was established that this combination is sufficient and strong because the sorption and solubility is low and meets the normative requirements [22].

## 4. Conclusions

This thesis put forward at the beginning of this study has been confirmed. The addition of feldspar to PMMA increases the resistance of such obtained composite towards compressive forces. The addition of 30% silicate feldspar to methacrylic material increases its compressive strength by about 30%. The thermal analysis confirmed weak but existing bonding between the filler and polymer matrix. The developed teeth, to which feldspar has been added, had proper adhesion strength to the denture plate and, as such, could be used in prosthetic treatments wherever it is necessary to use teeth with a greater resistance to mechanical wear. Composite teeth obtained in this way do not have cytotoxic properties and have satisfactory visual appearance.

## Figures and Tables

**Table 1:** Compressive strength after 24 h of conditioning at 37 °C [MPa].

Sample No.	Cervical Part (Neat PMMA)-A [MPa]	Dentin Part (10 wt.% Feldspar)-B [MPa]	Enamel Part (30 wt.% Feldspar)-C [MPa]
1	108.9	117.2	153.5
2	97.4	113.8	164.4
3	114.0	120.6	160.7
4	111.0	119.1	161.6
5	106.0	121.5	158.4
AVG	107.5 ^AC,AB^	118.4 ^BC^	159.7 ^AC,BC^
SD	6.3	3.1	4.1

^AC,AB,BC^ Statistically significant value for *p* < 0.01, SD—standard deviation, AVG—average.

**Table 2:** Compressive strength after 7 days of conditioning at 37 °C [MPa].

Sample No.	Cervical Part (Neat PMMA)-A [MPa]	Dentin Part (10 wt.% Feldspar)-B [MPa]	Enamel Part (30 wt.% Feldspar)-C [MPa]
1	98.2	107.3	133.6
2	92.4	98.8	144.5
3	97.8	101.5	150.6
4	101.0	102.5	148.7
5	91.3	110.6	158.1
AVG	96.1 ^AC^	104.1 ^BC^	147.1
SD	4.1	4.7	9.0

^AC,BC^ Statistically significant value for *p* < 0.01, SD—standard deviation, AVG—average.

**Table 3:** Tooth adhesion to denture base [N].

Sample No.	24 h [N]	7 Days [N]
1	29.43	28.81
2	24.52	26.43
3	29.04	26.12
4	28.36	25.75
5	26.66	25.76
AVG	27.60	26.57
SD	2.02	1.28

Not statistically significant value for *p* < 0.05 in both groups, SD—standard deviation, AVG—average.

## Data Availability

Not applicable.
